# Assessment of HIV prevalence among MSM in Tokyo using self-collected dried blood spots delivered through the postal service

**DOI:** 10.1186/s12879-018-3491-0

**Published:** 2018-12-05

**Authors:** Misao Takano, Kohta Iwahashi, Ikuo Satoh, Junko Araki, Takuya Kinami, Yuzuru Ikushima, Toshiya Fukuhara, Hiroo Obinata, Yasuyo Nakayama, Yoshimi Kikuchi, Shinichi Oka, the HIV Check Study Group, Kiyomi Tomonari, Hisahiro Sakuma, Yuko Sugino, Kazuko Ikeda, Mikiko Ogata, Kazuko Tanaka, Kiyoto Tsuchiya, Takahiro Aoki, Daisuke Mizushima, Yasuaki Yanagawa, Haruka Uemura, Masaki Nagai, Kazuhisa Mezaki, Kazuaki Terado, Seiichi Ichikawa

**Affiliations:** 1 Medical Genomic Center, National Center for Global Health and Medicine, 1-21-1 Toyama, Shinjuku-ku, Tokyo, 162-8655 Japan; 2 AKTA, 2-15-13-302 Shinjuku, Shinjuku-ku, Tokyo, 160-0022 Japan; 3 PLACE TOKYO, 4-11-5-403 Takadanobaba, Shinjuku-ku, Tokyo, 169-0075 Japan; 4 Higashi-Shinjuku Kokorono Clinic, 2F 6-28-12 Shinjuku, Shinjuku-ku, Tokyo, 160-0022 Japan; 5 AIDS Clinical Center, National Center for Global Health and Medicine, 1-21-1 Toyama, Shinjuku-ku, Tokyo, 162-8655 Japan

**Keywords:** HIV prevalence, MSM, Dried blood spot, Self-collection kit

## Abstract

**Background:**

Men who have sex with men (MSM) are at high risk of HIV infection. However, there are only few data on HIV prevalence in MSM in Japan. The objective of this study was to explore the HIV prevalence in MSM at Shinjuku 2-chome, a well known gay quarter in Tokyo.

**Methods:**

MSM directly collected the dried blood spot (DBS) self-collection HIV test kit from a drop-in center in Shinjuku 2-chome between August 2015 and December 2016. The participants collected their own blood by finger-prick and anonymously posted the kit to the laboratory. The participants accessed the study website and checked the results of their tests using unique ID and password. DBS was soaked in phosphate buffered saline overnight and the eluted sample was examined by the fourth generation HIV Ag/Ab test of LUMIPULSE (FUJIREBIO INC.), and followed by HISCL (Sysmex Corp.) when the first assay was positive. The result was defined provisionally positive if both were positive.

**Results:**

A total of 1702 HIV test kits were distributed and 1403 DBS were returned (return rate: 82.4%). Since 20.2% of participants collected the test kit more than once, the estimated number of actual test kit users was 1120. Based on the results of the test kit, 34 cases were provisionally diagnosed with HIV. The estimated prevalence was 3.04% (95% confidence interval: 2.03–4.04). Of these 34, 24 (70.6%) were later confirmed to be HIV-positive in the hospital, while the remaining 10 were lost to follow-up. Among the participants, 34.5% received HIV test for the first time. Especially in those aged 20–29, 46.0% were first time HIV testers.

**Conclusions:**

The prevalence of HIV infection in the study population was 3.04%. The high collection suggested a demand for this type of testing in MSM. The test should be expanded further to difficult-to-reach or hidden populations.

**Trial registration:**

This study was registered with the University Hospital Medical Information Network Clinical Trial Registry in August 20th, 2015 (Registry number: UMIN000018699).

**Electronic supplementary material:**

The online version of this article (10.1186/s12879-018-3491-0) contains supplementary material, which is available to authorized users.

## Background

According to the current Japanese national surveillance data, about 1500 new cases of HIV infection are recorded every year during the current decade, and of these, approximately 30% are registered in Tokyo [1]. The population at highest risk of HIV infection in Japan, like the rest of the world, is men who have sex with men (MSM) [1]; accounting for more than 70% of new cases.

HIV testing in Japan is free of charge and anonymous at public health centers. Over 450 public health centers provide the HIV test once a week or once a month at each site. Only one public HIV testing center (Minami Shinjuku Voluntary Counseling Testing center) opens every day throughout the year in Japan [2]. About 50% of the public health centers provide HIV testing during the daytime on weekdays and 30.8% do not provide the rapid test. Of the total, 72% of the public health centers require appointment for HIV testing [3]. The number of HIV tests conducted at the public health centers has recently been decreasing, from about 177,000 tests in 2008 to 118,000 tests in 2016 [4].

In addition to the inconvenience of the HIV test, the social stigma on homosexuality in general and discrimination against MSM and/or HIV infection is one of the reasons for HIV testing barriers. According to an internet survey conducted in 2016 to determine the reasons for why 2609 MSM had never received HIV test, 63.3% of these individuals had no opportunity, 31.0% were scared to know the result, 28.5% had considered no chance of HIV infection, 26.9% did not know the place of testing sites, and 15.9% felt uneasy explaining their sexuality [5].

Interestingly, however, the number of postal HIV testing has increased steeply from about 26,000 tests in 2005 to 91,000 tests in 2016 [3]. At present, 13 private firms sell the postal HIV test kits in Japan through the internet for around USD 20–40 [3]. However, postal HIV testing has not yet been approved in Japan mainly because it is based on the use of dry blood spot (DBS) in the HIV testing kit, a test technique that is yet not approved by the health authorities. In addition, postal HIV testing is provided without counseling. People with a positive HIV screening test are advised to visit the hospital or public health center for confirmatory test [3]. About 150 HIV screening test positive cases were identified by HIV postal tests in 2016; however, the number of clients who were linked to HIV care and treatment was not clear [3]. HIV care and treatment are fully covered by the public health insurance scheme in Japan.

One study showed that half of the postal HIV testing users in Japan were probably commercial sex workers [6]. Another study reported that rate of MSM was only 7.4% among the postal HIV testing users [7].

Based on this background, an HIV testing program that targets MSM using postal DBS and self-collection kit has been established and termed “HIV check”. The “HIV check” kits were distributed to MSM aged 20 years and older who were able to read Japanese in Shinjuku 2-chome gay town that has more than 400 commercial recreational facilities (bars, clubs, shops, and bathhouses). The primary objective of the present study was to document the prevalence of HIV in MSM, using the “HIV check”, who gather at Shinjuku 2-chome, a well known gay quarter with a considerable number of Tokyo’s MSM. The secondary objectives were to document the number of MSM who use the test, the number of the first-time HIV testers, and the rate of HIV-positive people linked to HIV care.

## Methods

### Study design

The “HIV check” kit was distributed between August 2015 and December 2016. The eligibility criteria were: (1) Men who reported sex with men; (2) aged ≥20 years; and (3) able to read Japanese. The target number of kits to be distributed was 1000 kits/year. This program was provided free of charge and anonymous, including the confirmatory test.

The participating institutions in the “HIV check” cooperative study included the AIDS Clinical Center (ACC), Higashi-Shinjuku Kokorono Clinic (HSKC) and two gay-friendly community-based organizations (CBO) in Tokyo, Japan. The first CBO, named AKTA, promoted “HIV check” and distributed the test kits to gay and bisexual men. The second CBO, named PLACE TOKYO, provided peer counseling at the distribution site and telephone counseling services. The ACC was the main laboratory for the HIV test using DBS. The ACC and HSKC accepted HIV positive cases by DBS and collected blood samples for the confirmatory test (HIV RNA test), and provided notification of the results and HIV care and treatment or referral to other medical facilities selected by the patients for regular consultations after the test. The ACC was also the main organizer of the entire “HIV check” study.

### Preparedness and management of this study

The “HIV check” kit was distributed by the staff of AKTA, which promotes HIV prevention and testing among MSM. Before the study implementation, lectures were given to the distribution staff on the method of HIV test by DBS, the necessary blood volume, attention points on blood collection and the meaning of the testing results. A procedure manual was also prepared and verified on how to confirm eligibility criteria, obtain informed consent, request questionnaire, distribute the testing kit, introduce to a peer counselor and maintain confidentiality obligation. The test kits were distributed by 6 staff members. All staff members including the distribution staff, peer counselors, medical staff and researchers, held a progress meeting once a month. Site visit monitoring was conducted regularly by a researcher from the AIDS Clinical Center.

### Recruitment of participants

Recruitment was achieved mainly through advertisements posted on mobile dating applications for MSM. A click on the banner advertisement took the interested individual to the study website of “HIV check”. In addition, the same advertisements were distributed to gay bars, gay shops, and bathhouses and published on the back cover of several gay magazines.

### Distribution of “HIV check” kit

The distribution of HIV test kits was conducted every Thursday night from 7 to 10 PM at the drop-in center (AKTA) in Shinjuku 2-chome gay town. Eligible men were enrolled in the study after they read the informed consent form and checked the agree box about the condition of anonymity. The participants also completed a short questionnaire. AKTA staff members explained how to use the self-collection kit and handed it directly to the participant. Furthermore, the PLACE TOKYO provided peer counselors at the same place and time when required.

The testing kit included lancets, filter paper, an instruction sheet, a booklet of related information (interpretation of results, telephone support lines), a unique ID and password and an envelope for posting the DBS to the ACC laboratory. The study participants took the testing kit to their homes, collected their own blood by finger-prick, and then posted the DBS to the ACC laboratory. AKTA is located in the center of Shinjuku 2-chome gay town. Some people come to take the HIV test kits with their friends. To maintain confidentiality, we asked them to take the test kit to their home.

### Questionnaires

After obtaining informed consent, a self-administered short questionnaire was distributed at AKTA. The questionnaire was developed for this study and included 1) residence prefecture, 2) age, 3) HIV testing history, 4) date of the last HIV testing and site, 5) frequency of obtaining the “HIV check” kit, 6) information source on “HIV check” and 7) reasons for selection of “HIV check” (Additional file 1: Table S1). The questionnaire was completed anonymously and placed upon completion into a collection box at the distribution site by the study participant. One questionnaire survey was completed each time the participant obtained a testing kit. The questionnaire survey was conducted independent of the HIV test and was not linked to the testing results. One member of the research team periodically collected the questionnaire and entered the responses into the database.

### Method of HIV testing

A paper disc of 5.5 mm diameter was cut out from the dried blood spot on the filter-paper (Transport blood collection filter paper set, EIKEN CHEMICAL CO, Tokyo, Japan), and soaked in 600 μL phosphate buffered saline before placing it in a vortex mixer for 5 s. Blood was eluted overnight at 4 °C. The eluted sample was centrifuged at 3000 rmp for 10 min and 200 μL of the supernatant was used for the first HIV test. The amount of blood from DBS in this assay was equivalent to 3 μL of serum sample (Additional file 2: Figure S1).

The first HIV test was conducted in the ACC laboratory using LUMIPULSE®S (FUJIREBIO INC. Japan, Chemiluminescence Enzyme Immunoassay, the fourth-generation antigen/antibody HIV test), and the procedure supplied by the manufacturer. The cut-off index for the first reading was ≥1.0. If the first reading was positive, the sample was forwarded to the second HIV test by HISCL®-5000 (Sysmex Corp. Japan, Chemiluminescence Enzyme Immunoassay, the fourth-generation antigen/antibody HIV test) in the central laboratory of the Center Hospital of National Center for Global Health and Medicine. This laboratory undergoes regular quality assurance. The cut-off index of the second reading was ≥1.0. When the second test was also positive, the case was defined as provisionally positive.

Before this study, the assay was assessed to determine the intra-assay (Additional file 3: Table S2A) and inter-assay variability (Additional file 3: Table S2B), sensitivity analysis by diluting positive samples (Additional file 3: Table S2C), comparison of eluted DBS samples and amount of serum (Additional file 3: Table S2D). Carry over assessment by cutting out DBS was repeated ten-times and no carry over results was detected. Repeating the entire procedures including postal step were conducted at day 4, 9, and 15 with 3 positive samples. We obtained positive results in all experiments.

### Definition of HIV positive test

The Japanese Guideline for HIV testing considers the result positive if both the screening test and confirmatory test by western blot or HIV RNA are positive. In this study, we used two kinds of the fourth generation HIV Ag/Ab tests. The test was considered HIV positive when both assays were positive. However, a banner was used to display this message: “You need a confirmatory test” on the web page of the group and advised the patients with both positive test to visit the hospital. If the first assay (LUMIPULSE) was positive and the second assay (HISCL) was negative, we also displayed “You need confirmatory test” and advised to visit hospital for further confirmation.

### Study flow

HIV test was conducted within a couple of days after receiving DBS. The participants accessed the study website and checked their results using their unique ID and password. On that website, the test results were displayed as follows; 1) You need a confirmatory test, 2) HIV negative, 3) insufficient sample. Participant reading “You need a confirmatory test”, made an appointment to visit the HSKC or ACC for the confirmatory test through the same website (Additional file 4: Figure S2), or were provided with a referral letter from the website to visit any other hospital of their choice.

### Statistical analysis

The questionnaire was completed in a completely anonymous manner. The results of HIV test and questionnaire were not linked. Data of the questionnaire were analyzed descriptively. The “HIV check” kit was provided several times during the study period. Therefore, the participants answered the questionnaire each time. The characteristics of the participants were extracted from the first-time response questionnaire. The prevalence in this study was calculated based on the number of two positive fourth generation HIV Ag/Ab tests. The prevalence was calculated with 95% confidence interval.

## Results

During the study period, a total of 1702 HIV testing kits were distributed to MSM. Of these, 1403 DBS were posted to the laboratory (return rate of 82.4%)**.** Thirty-four cases were positive by the two fourth generation HIV test kits, and thus were diagnosed as HIV-positive. Three cases were LUMIPULSE-positive but HISCL-negative. These 3 individuals later visited our hospitals and were confirmed to be HIV-negative based on HIV RNA negative. In other words, these three were false positive. Furthermore, 1358 cases were negative, including the three false positive cases. It was not possible to evaluate the presence of the false negative cases because we could not link repeated results due to anonymous manner of this study. The samples provided by 11 cases were considered insufficient; the DBS was too small to be used for the HIV test.

**Fig. 1:**
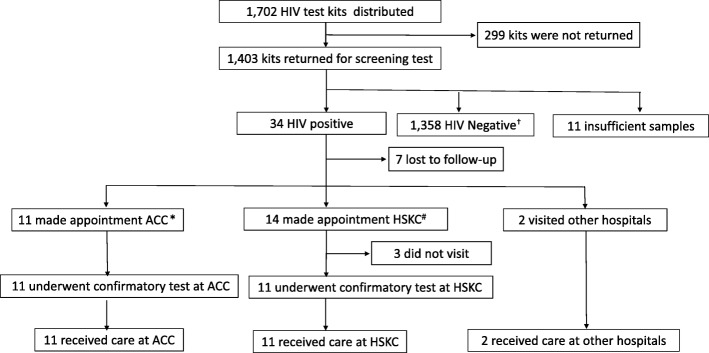
Flow diagram of the study and results. ^†^1358 include 3 false positive subjects who were LUMIPULSE-positive, HISCL-negative and HIV RNA-negative. ^*^ACC; AIDS Clinical Center, ^#^HSKC; Higashi-Shinjuku Kokorono Clinic

Of the 1403 participants, 1392 (99.2%) accessed the website for the test results. Among the 34 provisional HIV-positive cases, 22 visited the HSKC and ACC to undergo HIV RNA and were confirmed to have HIV infection, 2 cases were diagnosed at other hospitals, while 10 cases were lost to follow-up and no information is available on whether they visited another hospital or public health center because to the anonymous nature of the study. Thus, a total of 24 HIV-positive cases (70.6%) were referred for further clinical care and treatment among the 34 provisionally-diagnosed cases (Fig. 1).

We distributed 1702 questionnaire and collected all of them though a few were blank. The frequency of receiving the “HIV check” kit is shown in Table 1. About 20% of the participants received the HIV test kit more than once. The characteristics of the participants who received the test kit for the first time are summarized in Table 2. As to the age of the participants who received the test kit, the proportion of individuals aged 20–39 years was 74.8%. About 35% of the participants had never had an HIV test in the past. Especially in those aged 20–29, 46.0% were first time HIV testers. Furthermore, only 23.3% of the study subjects reported undergoing HIV test in the preceding year. The majority of the MSM (68.1%) lived in Tokyo. Table 3 lists the HIV test sites previously visited by participants for HIV test. This question had been included in the questionnaire since March 2016; therefore the response number was 510. Among the MSM who had undertaken the HIV test previously, 64.3% used public health centers or a Minami Shinjuku VCT center previously. Only 3.5% used the commercial postal HIV test before participating in this study.

**Table 1:** Frequency of receiving the “HIV check” kit

Frequency	*n* = 1702	%
Once	1358	79.8
Twice	247	14.5
Three times	68	4.0
Four times or more	26	1.5
Unknown	3	0.2

**Table 2:** Demographic characteristics of MSM who received “HIV check” kits

Characteristics	*n* = 1358^a^	%
Age group^b^:		
20–29	556	40.9
30–39	460	33.9
40–49	291	21.4
50-	50	3.7
Unknown	1	0.1
HIV testing history		
Never tested	469	34.5
Tested at least once	888	65.4
Within 1 year	317	23.3
1–2 years previously	346	25.5
Over 3 years previously	209	15.4
Test date unknown	16	1.2
Unknown	1	0.1
First HIV test ever according to age		
20–29	256/556	46.0
30–39	114/460	24.8
40–49	80/291	27.5
50-	18/50	36.0
Unknown	1	–
Residence prefectures		
Tokyo	925	68.1
Kanto (around Tokyo area)	386	28.4
Other	47	3.5

^a^Numbers represent individuals who received the “HIV check” kit for the first time

^b^The minimum age for eligibility was 20 years

**Table 3:** The latest HIV test sites

Test site	*n* = 510*	%
Public health center	241	47.2
Minami Shinjuku VCT^†^ center	87	17.1
Hospital	72	14.1
Clinic	60	11.8
Postal blood sample self-collection test	18	3.5
Other	22	4.3
Unknown	10	2.0

*This question was carried out since March 2016. Therefore, the number of subjects is 510 only rather than 888

^†^VCT: Voluntary counseling and Testing Services

**Fig. 2:**
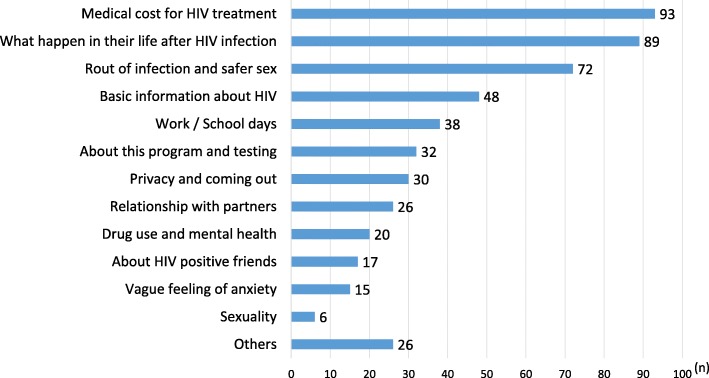
Questions asked by the 232 participants at peer counseling

The number of MSM who underwent counseling after receiving the HIV test kit was 232 (17.1%). The most frequently requested information was the medical cost of HIV treatment, followed by how HIV infection would change their life (Fig. 2).

Among the 1702 individuals who received the HIV test kits, 1403 DBS were returned. Since 20.2% of the participants obtained the test kit more than once, the estimated number of actual test kit users was 1120. Thirty-four subjects were positive for both LUMIPLUSE and HISCL. If all the 34 cases were truly positive, the estimated prevalence of HIV in MSM was 3.04% (95% confidence interval: 2.03–4.04) in this study.

## Discussion

The prevalence of HIV in this study was 3.04%, which is similar to that reported in a previous study of 423 MSM (3.1%) in a community-based center in Yokohama city close to Tokyo [8]. The result of this study is considered the true prevalence in MSM in the largest gay town in Tokyo, where HIV infection is the most concentrated in Japan. The above prevalence can be used as an index in any evaluation of HIV status and preventative measures before the forthcoming Tokyo Olympic Games in 2020.

In this study, 1702 HIV test kits were distributed to MSM in Tokyo and 1403 DBS were returned (return rate: 82.4%). Among the latter, 99.2% accessed the web to check the results. A few studies have reported that the DBS self-collection test was not preferred to the oral fluid rapid self-test or medical facility/community HIV testing [9, 10]. Our study suggests that the self-collected HIV testing kit from the drop-in center located within a gay town is potentially suitable for HIV testing of MSM.

Among the participants in this study, 34.5% received HIV testing for the first time. Especially in MSM in their twenties, nearly half of them received HIV test for the first time. This indicates that the HIV test kit is an acceptable method for such subjects, despite the need for self-collection of blood samples through a finger-prick. In addition, among the study participants, only 3.5% had used the commercial postal HIV testing before participating in this study. In Japan, the number of people using commercial postal HIV testing is increasing [3], we believe that the number of MSM users of this service is still limited. Efforts to promote postal testing among MSM could enhance the use this service and complement the test in public health centers.

The present study confirmed that DBS, postal, and self-collection HIV testing are useful tool to expand the reach to MSM. For example, it provides ease-of-access for MSM who live in rural areas, where homosexuality is still a stigma and discrimination against MSM and/or HIV infection is still strong. With regard to confidentiality, a self-test with oral fluid was also reported to have merit of nondisclosure of sexual orientation to healthcare providers, similar to the self-collection test [11]. At present, a large number of illegal migrants have settled in Japan, and some have arrived from HIV endemic countries and potentially high-risk population. The availability of our website in other languages should allow such people access to the HIV testing.

Our study showed that 24 out of the 34 HIV-positive patients (70.6%) sought clinical care. The finding is comparable to the rate of public health centers (78.8%) [12]. In Japan, it was reported that almost all HIV-positive patients consult clinical facilities and seek treatment; only 1.2% of HIV-positive patients did not visit hospitals after the diagnosis of HIV infection [13]. In the present study, we could not confirm that 10 HIV-positive individuals received confirmatory test and sought clinical care. However, we are hopeful that they visited other hospitals to receive clinical care and treatment.

The design of the present study included the services of a counselor at the time of the kit distribution. About 17% of the study participants requested counseling when they received the HIV test kit. However, when this program is expanded nationwide, it will be difficult to include counselors at all HIV testing kit distribution sites. Other counseling methods, such as live-chat consultation on the website [14] or telephone counseling would be necessary.

Previous reports described the benefits of home-based oral fluid rapid HIV self-testing in increasing the number of tests [15, 16]. However, it is also reported that the sensitivity of such tests is lower than that of blood tests [17–19]. In the present study, we selected DBS because the sensitivity of the DBS using HIV Ag/Ab test is reported to be similar to that of the serum test [20–22].

One limitation of this study is the lack of information on the sexual behavior of the participants. Future studies should include the sexual behavior of the participants. As a clinical study in Japan, the participants were limited to subjects older than 20 years of age. Adolescents are sexually active and hard to reach population. The postal HIV testing should be expanded to the younger population in the future.

## Conclusions

The estimated prevalence of HIV infection among MSM was 3.04% in this study. The high uptake suggested that there is a demand for this type of testing in MSM. The testing can be potentially expanded to difficult-to-reach or hidden populations.

## Additional files

Additional file 1: **Table S1.** Self- administered questionnaire. (DOCX 45 kb)

Additional file 2: **Figure S1.** Diameter of dropped whole blood on filter paper. Number in the figure means amount of whole blood dropped. 5.5 mm diameter of filter-paper contains 6 μL of whole blood. It is equal to 3 μL of serum sample. (PPTX 178 kb)

Additional file 3: **Table S2A.** The intra-assay valiability. **Table S2B.** The inter-assay valiability. **Table S2C.** Sensitivity analysis with diluted samples. **Table S2D.** Association of C.O.I using eluted DBS and serum sample. (ZIP 31 kb)

Additional file 4: **Figure S2.** Link to clinical care and treatment through the study website. If the result of the HIV test was positive, the subject was able to set up an appointment at a clinic through the same website to receive a confirmatory test and treatment. (PPTX 688 kb)

## Abbreviations

ACC: AIDS Clinical Center
CBO: Community-based organization
DBS: Dried blood spot
HSKC: Higashi-Shinjuku Kokorono Clinic
MSM: men who have sex with men
UMIN-CTR: University Hospital Medical Information Network Clinical Trial Registry
