# Supplementary material for: Assessment of HIV prevalence among MSM in Tokyo using self-collected dried blood spots delivered through the postal service
Source: BMC Infect Dis. 2018 Dec 5;18:627. doi: 10.1186/s12879-018-3491-0 (PMC6282288; doi:10.1186/s12879-018-3491-0)
Supplement: Supplementary file 1 — Table S1. Self- administered questionnaire. (DOCX 45 kb) [file 12879_2018_3491_MOESM1_ESM.docx]

Supplemental Table 1. Self- administered questionnaire

AKTA's staff will not see this questionnaire.　Please drop it in a questionnaire collection box after filling in

Q1. Which area do you live in?

1. Tokyo

2. Kanto (excluding Tokyo)→ □Kanagawa　□Saitama　□Chiba　□Ibaraki　□Gunma　□Tochigi

3. Other areas in Japan

4. Overseas

Q2. How old are you? years

Q3. Have you ever been tested for HIV exclude
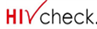
?

1. Yes　 2. No →　Q6

Q4. When was your last HIV test?

1.Within 1 year　→　Before　　　months　　2. 1-2 years ago 3. Over 3 years ago

Q5. Where/How did you undergo your last HIV test?

1. Public Health Center. 2. Hospital. 3. Clinic. 4. Minami Shinjuku HIV testing center.

5. Postal HIV test. 7. Other

The following questions are about
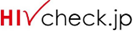
 which are provided in this research.

Q6. How many times have you received this
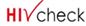
 kit, including this time?

1. This is the first time 　2. Second time. 3. Third time. 4. Fourth time or more

Q7. From where did you get information on
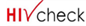
? (Check all that apply)

1. Gay bar. 2. Nightclub. 3. Event. 4. Group activities. 5. Bathhouse. 6. AKTA

7. Flyer at gay shop. 8. Dating apps. 9. Facebook/Twitter. 10. Gay magazine. 11. Friends

12. Others ( )

Q8. What triggered did you receive
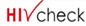
? (Check all that apply)

1. This is a regular HIV test

2. I made a new partner.

3. I had a risky sex

4. I had a sexually transmitted infection

5. Poor physical status

6. I had sex with HIV-positive person

7. “HIV check” was recommended by friends.

8. I knew HIV infected people around me

9. Others ( )

Q9. Why did you choose
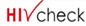
 as the HIV test? (Check all that apply)

1. I can take the test at my convenience

2. I do not have to go to the testing site

3. This test needs less time to meet someone

4. It is clear where I can receive a counseling if I am positive.

5. I think “HIV check” is a trustworthy test.

6. It is clear where I can seek consultation if I am positive

7. Recommended by friends and acquaintances

Q10. In this study,
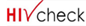
 is free. What is the maximum amount you'll be willing to pay in the future for a
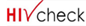
 kit? Yen
