# Supplementary figures and images for: Assessment of HIV prevalence among MSM in Tokyo using self-collected dried blood spots delivered through the postal service
Source: BMC Infect Dis. 2018 Dec 5;18:627. doi: 10.1186/s12879-018-3491-0 (PMC6282288; doi:10.1186/s12879-018-3491-0)

## Slide 1
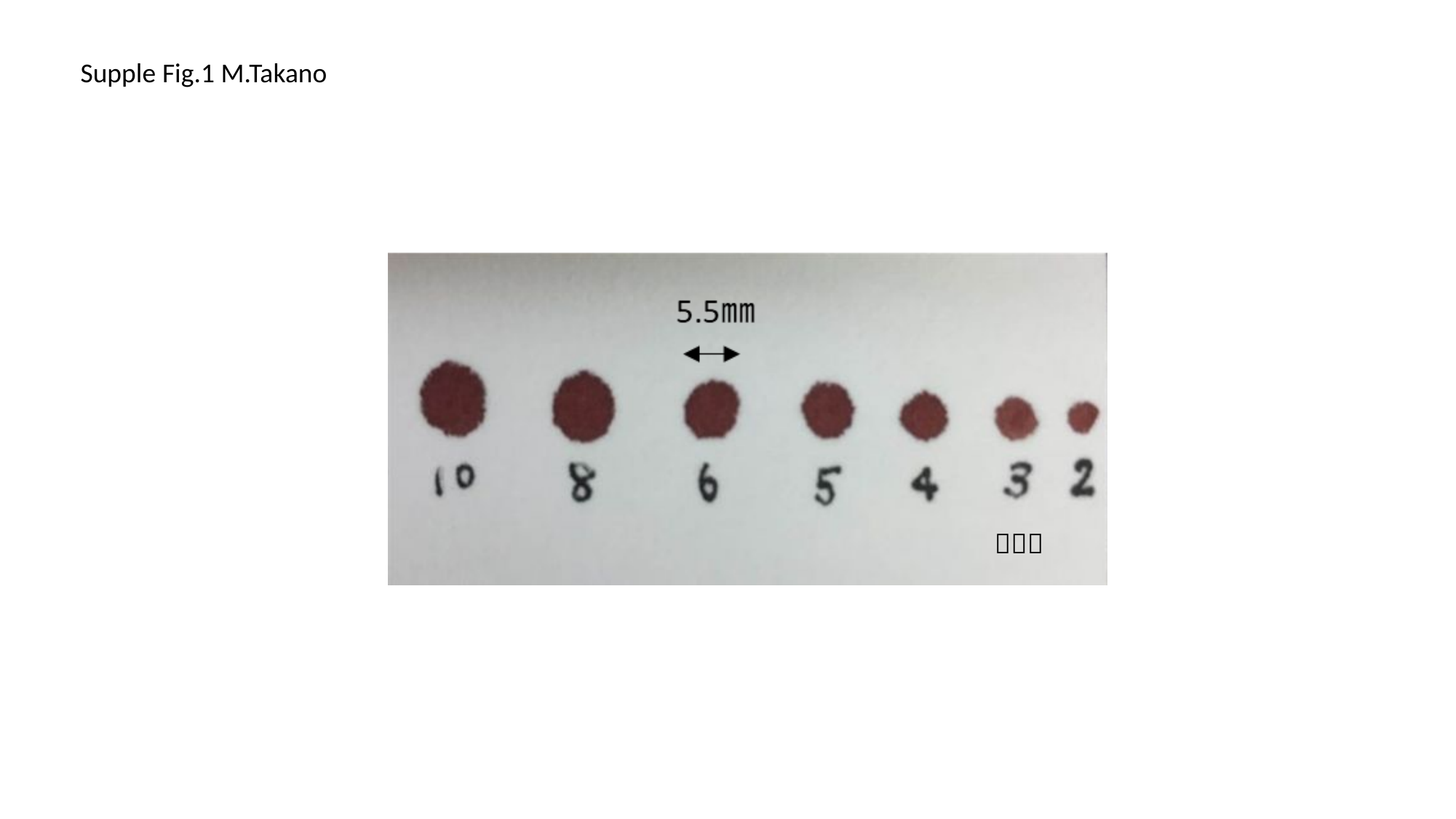

Supple Fig.1 M.Takano
（㎕）

Supplement: Supplementary file 2 — Figure S1. Diameter of dropped whole blood on filter paper. Number in the figure means amount of whole blood dropped. 5.5 mm diameter of filter-paper contains 6 μL of whole blood. It is equal to 3 μL of serum sample. (PPTX 178 kb) [file 12879_2018_3491_MOESM2_ESM.pptx]

## Slide 1
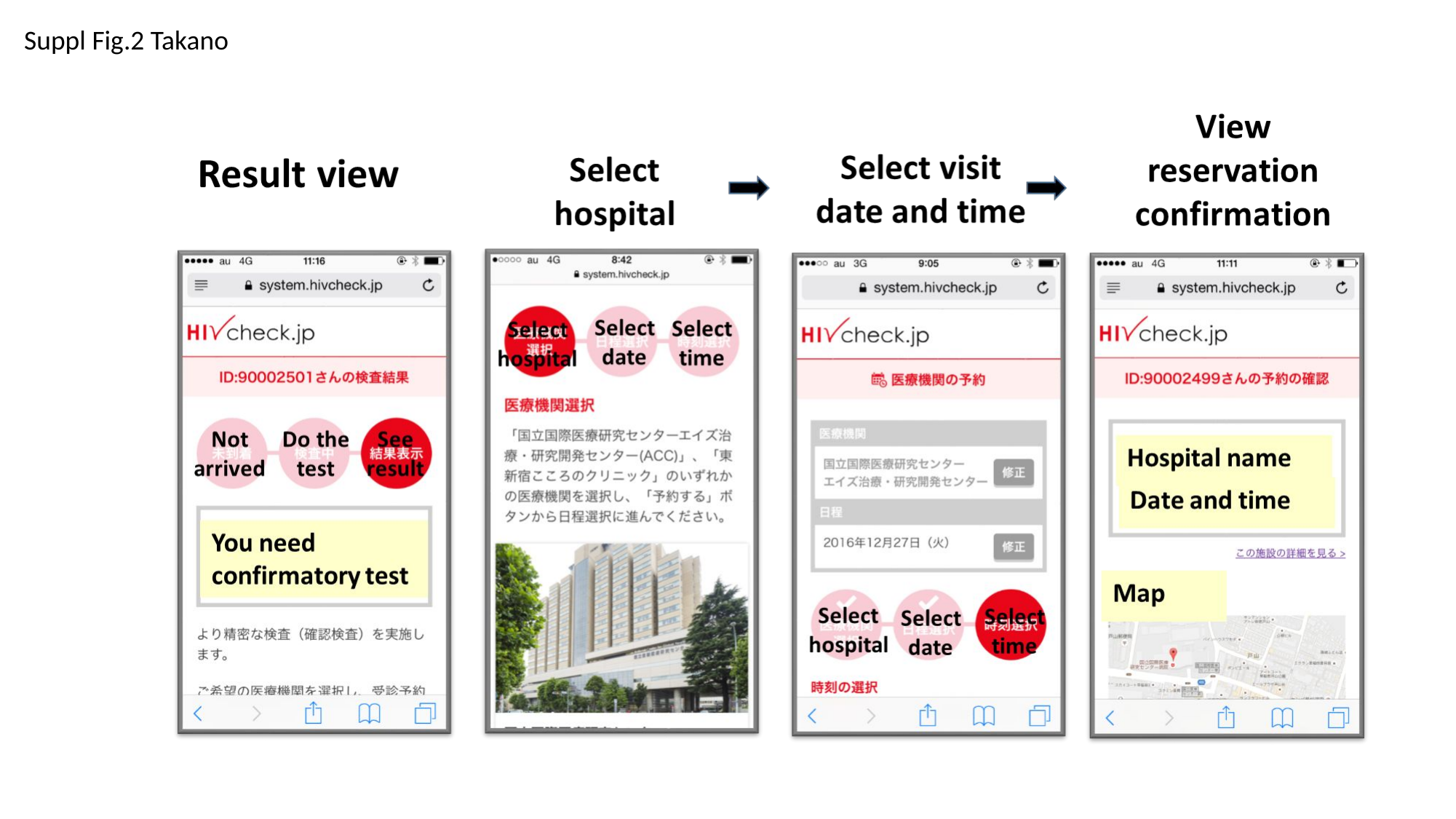

Suppl Fig.2 Takano

Supplement: Supplementary file 4 — Figure S2. Link to clinical care and treatment through the study website. If the result of the HIV test was positive, the subject was able to set up an appointment at a clinic through the same website to receive a confirmatory test and treatment. (PPTX 688 kb) [file 12879_2018_3491_MOESM4_ESM.pptx]
